# Physical injury assessment of male versus female chiropractic students when learning and performing various adjustive techniques: a preliminary investigative study

**DOI:** 10.1186/1746-1340-14-17

**Published:** 2006-08-24

**Authors:** Debra W Bisiacchi, Laura L Huber

**Affiliations:** 1 Division of Chiropractic Sciences, Life University College of Chiropractic, 1269 Barclay Circle, Marietta, GA 30060, USA

## Abstract

**Background:**

Reports of musculoskeletal injuries that some chiropractic students experienced while in the role of adjustor became increasingly evident and developed into the basis of this study. The main objective of this study was to survey a select student population and identify, by gender, the specific types of musculoskeletal injuries they experienced when learning adjustive techniques in the classroom, and performing them in the clinical setting.

**Methods:**

A survey was developed to record musculoskeletal injuries that students reported to have sustained while practicing chiropractic adjustment set-ups and while delivering adjustments. The survey was modeled from similar instruments used in the university's clinic as well as those used in professional practice. Stratified sampling was used to obtain participants for the study. Data reported the anatomical areas of injury, adjustive technique utilized, the type of injury received, and the recovery time from sustained injuries. The survey also inquired as to the type and area of any past physical injuries as well as the mechanism(s) of injury.

**Results:**

Data obtained from the study identified injuries of the shoulder, wrist, elbow, neck, low back, and mid-back. The low back was the most common injury site reported by females, and the neck was the most common site reported by males. The reported wrist injuries in both genders were 1% male complaints and 17% female complaints. A total of 13% of female respondents reported shoulder injuries, whereas less than 1% of male respondents indicated similar complaints.

**Conclusion:**

The data collected from the project indicated that obtaining further information on the subject would be worthwhile, and could provide an integral step toward developing methods of behavior modification in an attempt to reduce and/or prevent the incidence of musculoskeletal injuries.

## Background

Due to the physical requirements of their jobs, healthcare professionals can be susceptible to various physical injuries. A review of the literature, abstracts, bibliographies, and computer databases revealed numerous studies investigating the prevalence of musculoskeletal injuries in certain high-risk groups [1-3]. In a study conducted by Molumphy et al., of 344 physical therapists, 29% reported work-related low back pain [4]. French et al. found that 80.9% of 47 acute-care nursing staff reported the occurrence of some form of low back pain during their careers [5]. A study by Lehto et al. indicated that in 131 active dentists, 37% experienced pain and/or disability in the low back for the previous year, and 42% experienced neck and shoulder problems [6]. From a review of the literature, Morse et al. concluded that there was a prevalence of musculoskeletal symptoms in 63% to 93% of dental hygienists [7]. Hignett summarized findings from over 80 published studies regarding work-related musculoskeletal dysfunction, and concluded that nursing appeared to be a high risk occupation with respect to low back pain [8].

The literature review also revealed some studies that addressed specific musculoskeletal injuries sustained by chiropractors. Homack's survey of 69 chiropractic respondents revealed that the anatomical structures most at risk for injury were the low back, shoulder, and wrist. Patient handling and delivery of side-posture procedures were identified as the activities most frequently resulting in those injuries [9]. Rupert and Ebete found that of 451 surveyed chiropractors, 57% reported work-related musculoskeletal injuries during their careers [10]. Mior and Diakow's epidemiological survey of 320 Canadian chiropractors found the overall prevalence of back pain was 87%, and that low back pain was predominant in 74% of the responding chiropractors [11]. In a survey of practicing chiropractors conducted by Holm and Rose, most reported injuries were classified as soft tissue, and had occurred while either performing or positioning a patient for "manipulation" [12].

Further review of the literature revealed numerous biomechanical studies suggesting that common mechanisms of injury in musculoskeletal disorders included bending, lifting, pulling, and sustained awkward postures. Occupational tasks that increased the magnitude of trunk velocity and sagittal angle were found to significantly increase the risk of injury [13,14]. Many adjustive techniques require the chiropractor to maintain awkward postures such as stooping, bending, and rotating at the same time forces are exerted. Chiropractors are subject to these dynamic motions on a continual basis and can experience unacceptable levels of spinal loading [15]. This occurrence can make them more susceptible to injury.

In the training of chiropractic professionals and other health care providers, some type of physical exertion and repetition is expected. It is important to identify musculoskeletal stresses and the mechanisms of injury. Nyland and Grimmer investigated the prevalence of low back pain in physiotherapy students [16]. Jackson and Liles also addressed working postures in this particular student population [17]. Since technique courses are a required part of the chiropractic curriculum, they necessitate that students develop psychomotor skills [18,19] as well as strength and agility [20].

At Life University's College of Chiropractic, students analyze and adjust under direct doctor supervision in both the classroom and the clinical setting. In the classroom lab setting, students learn to position themselves to deliver, and position their patients to receive, chiropractic adjustments. They also learn to thrust directly into the spinal areas of their classmates. In the clinical environment, students deliver adjustments to their peers, to undergraduate students and, in upper quarters, to the general public. In order to master these skills, students are required to perform repetitive adjusting procedures, but may not have the necessary strength or skills to withstand sustaining some type of musculoskeletal injury.

Reports of these injuries that some students experienced in the role of adjustor became increasingly evident and eventually developed into the basis of this study. The main objective of this study was to survey a select student population and identify, by gender, the particular types of musculoskeletal injuries experienced when learning adjustive techniques in the classroom, and performing them in the clinical setting.

## Methods

With the approval of the Life University's Institutional Review Board, a survey was developed [see Additional file 1] to record musculoskeletal injuries that students reported to have sustained while practicing and delivering chiropractic adjustments. The survey was modeled from similar instruments used in the university's clinic as well as those used in professional practice, and was not subject to initial peer review.

Since specific information was needed, the participants were given a set of choices for each question, rather than being asked open-ended questions. Some of the participant demographic information requested was gender, age, height, and weight. Other information requested was the anatomical area of injury, adjustive technique utilized, the type of injury received, and the recovery time from sustained injuries. Inquiries were also made as to the type of past physical injuries that participants experienced and the mechanism(s) of injury. Some of the collected demographic data that was not specifically used in this study was allocated for future studies.

Stratified sampling was used to obtain participants for the study. Chiropractic students who were enrolled in 2^nd ^to 4^th ^year of study were asked to complete the survey to ascertain any injuries they may have sustained while practicing or performing chiropractic adjustments. Students who had been taught adjustive procedures, and interns who were performing supervised adjustments in the clinics, were included in the pool of participants. Excluded from the study were students who were not enrolled in the College of Chiropractic, and those who had not completed any chiropractic technique courses at the time the survey was distributed.

Although the study was designed to target and survey all qualified students, several obstacles prevented this from occurring. The survey required about 10–15 minutes time to complete, which prevented its distribution in short, 1-hour lecture courses. Also, it required a cooperative effort from instructors, which limited survey distribution. No surveys were distributed in the clinics in order to avoid duplication of those done in the classroom. There was a delay in the design of the survey instrument, which also contributed to time constraints. Therefore only 150 of the 378 eligible students were actually surveyed.

Prior to the distribution of the survey, the authors described to the eligible participants the purpose and the intent of the study. Since student identification numbers were required, the participants were informed that the survey was confidential and that the information would be stored in a secured location. Students were also told that they had a right to withdraw from the study at any time. Each was then required to complete and submit an informed consent form and retain a copy for his or her records.

Limited verbal instructions of how to complete the survey were given at the beginning of each distribution period. Once completed and collected, the surveys were submitted to university's Office of Institutional Effectiveness Planning and Research for compilation, analysis, and descriptive statistics of the data. The surveys were also reviewed manually for gross errors or misinformation.

## Results

**Figure 1:**
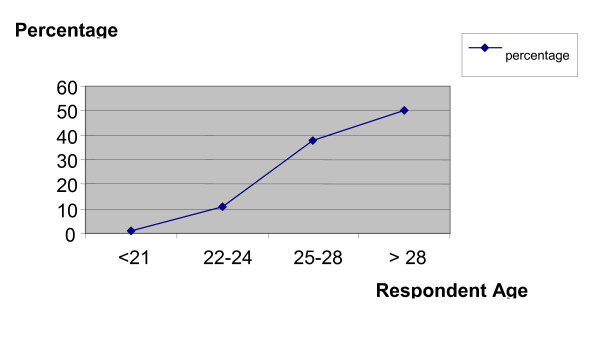
Participant Age and Response Data.

Of the 150 surveys that were distributed, 125 were returned, indicating an 83.3 % participant response rate. Not included were those surveys in which respondents reported injuries sustained as patients versus those sustained while delivering the adjustments. Not all questions were answered on all surveys, but available data was still collected. Differences in numbers and percentages in this study are reflective of the actual number of responses to each specific question that was asked. Of the useable responses received, 43 were from women, indicating an overall respondent rate of 36%, and 77 were from men, indicating an overall respondent rate of 64%. The data reported that the majority of respondents were in the 3^rd ^year of study (53 students, 44%). Participants ranged in age from under 21 years to older than 28 years. Only 1 student was younger than 21 years old (1%), 14 were aged 22 to 24 years (11%), 47 were aged 25 to 28 years (38%), and 61 were older than 28 years (50%) (Figure 1).

**Figure 2:**
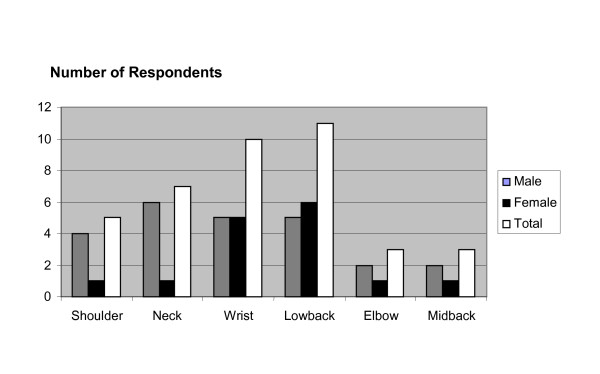
Anatomical Area of Injury.

Data obtained from the study reported injuries of the shoulder, wrist, elbow, neck, low back, and mid-back. (Figure 2). The low back was the most common injury site reported by females (19%), and the neck was the most common injury site reported by males (11%). The reported wrist injuries in both genders were 1% of male respondents and 17% of female respondents. A total of 13% of female participants reported shoulder injuries, whereas less than 1% of male participants indicated similar complaints.

**Figure 3:**
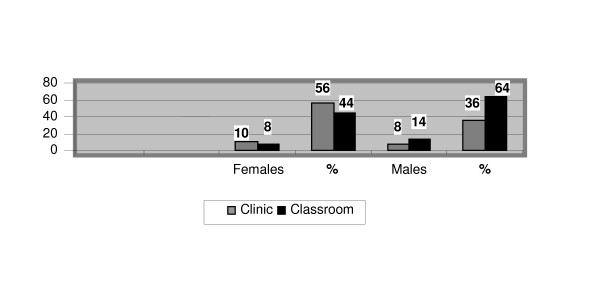
Setting of Injury Occurrence.

In 54% of the respondents, injuries were reported to have occurred in the learning lab environment, 64% to males and 44% to females. While in 46% of the respondents, 36% male and 56% female, injuries were reported to have occurred while performing adjustive techniques in the clinical setting (Figure 3). The data indicated that 60% of the injuries were reported to have occurred within the 6 months prior to distribution of the survey, 52% reported by men and 77% reported by women. In addition, 35% of the students, 31% of the male respondents and 42% of the female respondents, reported that they were still recovering from their injuries.

The adjustive techniques surveyed were those used at the college at the time of this study, and were limited to those addressing only the spine. Included were Full-Spine/Diversified side posture, supine and prone cervical set procedures, Thompson™ technique, Toggle technique, and Activator Methods™ technique. Appropriate analysis and protocol for each technique was a mandatory component for actual delivery of the adjustment, but was not utilized for simulated set-ups.

Students indicated on the surveys that performing Full-Spine side posture adjusting procedures was the most common mode producing their injuries. While performing these procedures, both male and female respondents reported low back and shoulder injuries. Supine cervical moves were the second most common procedure resulting in student complaints. These moves were reported to have produced wrist injury in many students. Prone cervical adjustments ranked third overall. The data suggested that these moves produced shoulder problems in female respondents and wrist problems in male respondents (Table 1).

**Table 1:** Reported Injuries per Technique

Adjusting Procedure	Injuries Reported	Male	Female
Side Posture	23	11	12
Prone	4	3	1
Supine Cervical	10	6	4
Seated Cervical	1	1	0
Activator™	1	1	0
Thompson™	2	0	2
Gonstead Chair™	0	0	0
Toggle:	2	2	0

## Discussion

In our search of keywords, several studies highlighting chiropractic injuries were available, but there were very few that addressed gender differences when delivering the particular therapy. In the Macanuel et al. studies, for example, most references were to injuries sustained by students while receiving adjustments, and gender references were made only with respect to actual responses received by participants [18,19]. As well, in studies by Sensted et al., injuries reported were those sustained when receiving versus delivering "spinal manipulative therapy" [21,22].

In some studies of work-related musculoskeletal symptoms in other professional students, dental hygiene students reported that 60% experienced some pain, 46% reported upper extremity pain, 13% reported numbness, and 13% reported white or painful fingers in cold temperatures [7]. Reports from another study by Anton et al. included a high prevalence of neck pain (68.5%) and shoulder symptoms (60%) in dental hygiene students [23]. Bork et al. reported that the highest annual prevalence of musculoskeletal disorders in physical therapists was in the low back, upper back, and neck, and that lower incidences of injury occurred in the shoulders, elbows, hips and thighs, knees, ankles and feet. The study found that more female therapists than male therapists had reported spinal, wrist and hand symptoms [24].

It takes time and effort on the part of the novice to learn the sophisticated and complicated skills necessary to perform a range of chiropractic adjustive techniques [9,11,18,19]. Not all students have the same levels of coordination, dexterity, or experience when learning psychomotor skills, and some may be more adept or physically developed [20]. Data gathered from this preliminary study indicated that students reported sustaining injuries in their attempts to deliver adjustments in both the classroom and clinical settings, and that gender differences existed relative to the anatomical areas of injuries and the adjustive techniques used at the time of injury occurrence.

This study's findings did not reproduce similar gender difference complaints as those found by Mior and Diakow. They reported a higher prevalence of thoracic spine pain and shoulder pain complaints among female chiropractors and more low back pain complaints among male practitioners [11].

In the study of physical therapist injuries, Nyland and Grimmer found that, of first year students, females reported a greater prevalence of low back pain. However, in succeeding years of study, they found that 1^st ^and 4^th ^year female students reported a greater preponderance of low back pain, where 2nd and 3^rd ^year male students reported a greater preponderance of low back pain [16].

It was difficult to compare chiropractic gender difference findings to other health related fields due to the predominance of females in the roles of dental hygienist and nurse for example, versus the male majority population in the chiropractic profession.

Limitations of the study became evident as the project progressed. Since the surveys were retrospective in nature, students were required to recall and document the circumstances of injuries that may have occurred many months prior. If students were surveyed sooner, for example, after the completion of each technique course, there may have been more accuracy in their recall and responses.

Another limitation was that, although data was collected for participant height and weight, the association of injury sustained to body type was not a focus of this study. This demographic data could be a consideration for future studies.

A third limitation of the study was that the survey did not address the descriptive characteristics associated with the students' injuries, or the length of time the injuries remained. No data was collected with respect to the amount of time that had elapsed between delivery of the procedure and the onset of student symptoms. Areas addressing descriptors such as throbbing, aching, numbness, tingling, deep, sharp, etc., and injury duration could be added to future studies for better data collection.

During the time of the study, student enrollment was greater for males than for females and was reflected in the numbers and percentages of the participant responses. It was understood that the sample student group was representative of the general student population of those who were taking/had taken adjustive technique courses, and those who were active in the clinics. The problem with this pilot group was the small number of students who actually participated in it. In order to acquire more substantial information, subsequent studies are currently being developed to address a significantly larger population of participants.

When the survey was developed, there was no peer review or test/re-test performed due to time constraints. This proved to be another limitation of the study, and may have caused some confusion for the participants. If feedback on the survey's appropriateness had been obtained, the confusion may have been avoided. Although lacking in some areas, the same survey will be used in the later studies to determine whether the statistical data and the study's limitations are repeatable, and if they exhibit consistency on a larger scale.

Further investigation into the details of participant responses and additional analysis of the demographic data may reveal if a predisposition exists for certain individuals to sustain injury, and/or if a particular anatomical area could be involved. Once additional data is integrated, the relationship, if any, of individual characteristics to anatomical areas of injury, and the use of specific adjustive techniques can be determined. This associative data may also serve as a data base for the development and integration of injury-prevention measures into technique coursework.

## Conclusion

Data from this limited study reported some of the most common injuries students experienced while adjusting at Life University's College of Chiropractic, and further classified them by gender, age group, time frame of occurrence, and techniques that were used when the reported injuries occurred. This information, as well as identification of the specific anatomical sites of injuries, can provide an integral step toward developing methods of behavior modification in an attempt to reduce and/or prevent the incidence of musculoskeletal injuries. The data collected from the project indicated that obtaining further information on the subject would be worthwhile. Supplemental studies are planned involving a larger population of inter-collegiate participants, with the goals of developing methods of injury prevention, contributing to research, and continuing the dialogue within the profession.

## Competing interests

The author(s) declare that they have no competing interests.

## Authors' contributions

Debra W. Bisiacchi developed the study, and Laura L. Huber initiated the concept of the study. Both authors participated in the design and coordination of the study, developed initial presentation and surveys, performed thematic analysis, developed tables and prepared, wrote, and approved the final manuscript.

## Supplementary Material

Additional file 1 Physical Injury Assessment Survey. Sample of questionnaire used for this study Click here for file
