# Supplementary figures and images for: Physical injury assessment of male versus female chiropractic students when learning and performing various adjustive techniques: a preliminary investigative study
Source: Chiropr Osteopat. 2006 Aug 24;14:17. doi: 10.1186/1746-1340-14-17 (PMC1560146; doi:10.1186/1746-1340-14-17)

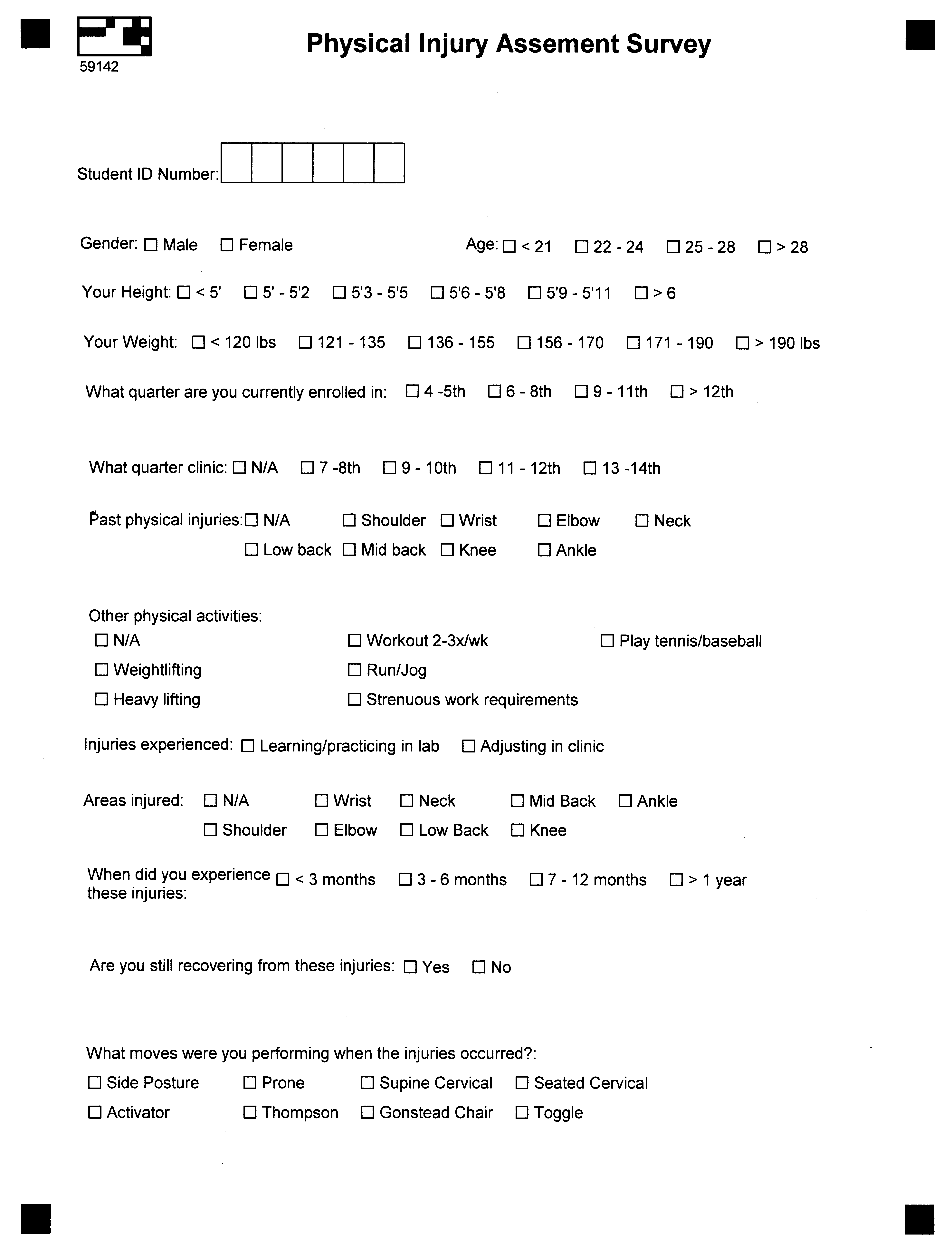

Supplement: Additional file 1 — Physical Injury Assessment Survey. Sample of questionnaire used for this study [file 1746-1340-14-17-S1.bmp]
